# Expanding the phenotypic spectrum of *LHCGR* signal peptide insertion variant: novel clinical and allelic findings causing Leydig cell hypoplasia type II

**DOI:** 10.1007/s42000-024-00546-x

**Published:** 2024-03-25

**Authors:** Heba Amin Hassan, Inas Mazen, Aya Elaidy, Alaa K Kamel, Noura R. Eissa, Mona L. Essawi

**Affiliations:** 1 Department of Medical Molecular Genetics, Human Genetics & Genome Research Institute, National Research Centre, 33 El-Bohouth street, Cairo, 12311 Egypt; 2 Department of Clinical Genetics, Human Genetics & Genome Research Institute, National Research Centre, Cairo, Egypt; 3 Department of Human Cytogenetics, Human Genetics & Genome Research Institute, National Research Centre, Cairo, Egypt

**Keywords:** LCH type II, WES, 33-bases insertion, TMD, 46,XY DSD

## Abstract

Leydig cell hypoplasia (LCH) type II is a rare disease with only a few cases reported. Patients presented with hypospadias, micropenis, undescended testes, or infertility. In this study, we report a new patient with compound heterozygous variants in the *LHCGR* gene and LCH type II phenotype. Methods: Whole exome sequencing (WES) was performed followed by Sanger sequencing to confirm the detected variants in the patient and his parents. Results: A novel missense variant (p.Phe444Cys) was identified in a highly conserved site and is verified to be in trans with the signal peptide’s 33-bases insertion variant. Conclusion: Our research provides a more comprehensive clinical and genetic spectrum of Leydig cell hypoplasia type II. It highlighted the importance of WES in the diagnosis of this uncommon genetic disorder as well as the expansion of the genotype of LCH type II.

**Supplementary Information:**

The online version contains supplementary material available at 10.1007/s42000-024-00546-x.

## Introduction

Luteinizing hormone/choriogonadotropin receptor *(LHCGR)* is involved in the development and maturation of male sexual organs both prenatally and postnatally [25]. Sexual differentiation is a critical and complex process which normally drives the development towards a consistent pathway with the chromosomal sex of the embryo. However, defects during prenatal development of male sex differentiation lead to abnormalities, ranging from mild defects such as micropenis or hypospadias to severe defects such as genetic males with female external genitalia [22]. *LHCGR* in fetal Leydig cells binds to placental chorionic gonadotrophin during the development of the male fetus to produce the testosterone required for male sexual differentiation, while *LHCGR* in the Leydig cells is stimulated postnatally by luteinizing hormone (LH) to produce testosterone essential for the development of male secondary sexual features, puberty, and spermatogenesis [3].

Leydig cell hypoplasia (LCH) is a rare autosomal recessive disorder (OMIM# 238,320) caused by inactivating variants of the luteinizing hormone/choriogonadotropin receptor (*LHCGR*) gene in genetic males. The incidence of LCH was estimated to be 1 in a million [7]. Patients with complete loss of *LHCGR* function are classified as type I, while type II LCH is a less severe type caused by *LHCGR* variants that preserve some receptor activation. The phenotype spectrum of LCH type II patients ranged from hypospadias or micropenis to undescended testes or infertility [22]. The milder form of LCH was initially described by Toledo and colleagues [30]. LCH type II patients presented with low testosterone levels unresponsive to exogenous hCG stimulation and with variable Leydig cell hypoplasia due to partial disruption of the *LHCGR* signaling and less severe receptor resistance [31]. Loss of function (inactivating) variants either in homo- or compound heterozygous patterns were reported to be associated with LCH type II [9, 34].

The human *LHCGR* gene is located on 2p21. It is one of the G protein-coupled receptors (GPCR), consisting of seven transmembrane domains (TMD) and a cytoplasmic C-terminal domain (ICD), which are encoded by the last exon of the gene (exon 11), whereas the first 10 exons encode for the signal peptide and extracellular domain (ECD) [2, 15]. Around 40 *LHCGR* variants with varying degrees of loss-of-function have been described which are distributed across all domains of the receptor.

In this study, we report a new case with compound heterozygous variants in the *LHCGR* gene. The phenotype of this case is entirely distinct from that of patients with the signal peptide 33-bases insertion variant which have previously been reported, and the second allelic variant was a novel missense in a highly conserved site. Therefore, our research provides a more comprehensive clinical and genetic spectrum of Leydig cell hypoplasia type II.

## Methods

### Clinical studies

A full history was taken from the patient, while he also underwent pedigree analysis, a thorough clinical examination, and genital examination including assessment of genital ambiguity according to Quigley et al. [23].

The patient was first referred to our center at the age of 9 years due to micropenis and undescended testis; follow-up until the age of 19 years old was established. He was born to consanguineous parents (second-degree cousins) with no family history of such medical conditions. Pregnancy and delivery were uneventful. General examination revealed no significant abnormality and anthropometric measurements were normal for his age.

The patient was investigated via chromosomal analysis, pelvic ultrasonography, and hormonal assessment of FSH, LH, testosterone, and ∆4-androstenedione. The patient’s gender was assessed using the psychological test of masculinity/femininity index.

### Molecular studies

Samples collection: Peripheral blood samples were collected from the patient and both parents on Vacutainer EDTA tubes. DNAs were extracted using a PAXgene DNA blood kit (Qiagen, Germany).

### Sanger sequencing

**Targeted gene sequencing** for the eight exons of the *AR* gene and the five exons of the *SRD5A2* gene were performed using previously reported conditions [13].

**Validation and confirmation of detected variants by WES:** Bidirectional sequencing of exons 1 and 11 of the LHCGR gene of the patient and parents was performed using the previously described protocol [6].

**Whole exome sequencing (WES):** Extracted DNA was measured using a spectrophotometer (Nanodrop 2000, Thermo Fisher Scientific, USA) and fluorimeter (DeNovix, Wilmington, USA). Qualified DNA was fragmented and the exome library was prepared and amplified. Exome sequence capture was performed using a SureSelect Human All Exon Kit (Agilent Technologies, USA), then sequenced using the NextSeq 550 platform according to the manufacturer’s protocol to obtain at least 20x coverage depth for > 98% of the targeted bases. Sequence reads were aligned to the human reference genome (GRCh37/hg19) and variants were called via Illumina DraGen germline pipeline. BaseSpace Sequence Hub was used for annotation and extensive filtration of variants. GenomAD and ClinVar were used to prioritize the variants depending on their frequency and clinical significance. The variants’ potential pathogenicity was determined using in silico analysis and confirmed using traditional Sanger sequencing. Due to their relative credibility in combining different tools, integrated ensemble prediction tools were used, e.g., REVEL and MetaRNN. Protein stability and structural effects due to non-synonymous variants were estimated using HOPE project, SDM, CUPSAT, and DUET tools [18–20, 33].

## Results

Genital examination revealed a penis-like phallus (2.5 cm), a single urethral opening at the tip of the phallus, a hypoplastic scrotum, palpable right testis in the inguinal region, and impalpable left testis.

Chromosomal analysis revealed 46,XY normal male karyotype. Pelvic imaging by ultrasonography showed that both testes were located in the inguinal canals and the prostate was visualized with no uterine shadow. Hormonal assessment showed basal (**testosterone** 0.2 ng/ml, and **∆4-androstenedione** 0.5 ng/ml) and post-HCG (**testosterone** 0.5 ng/ml, and **∆4-androstenedione** 0.6 ng/ml). Normal serum levels of **testosterone** and **∆4-androstenedione** in males were 3–12 ng/ml and 0.8–2.7 ng/ml, respectively.

The masculinity/femininity score indicated that the patient was classified as undifferentiated, but the personal interview showed that the patient had some masculine behavior and reevaluation at puberty was strongly recommended.

During follow-up at the age of 17 years, the patient reported that he had undergone orchiopexy surgery at the age of 13 years, and genital examination revealed a penile length of 4.5 cm, single urethral opening at the tip after correction of hypospadias, and both testes scrotal (left testis 12 ml and right testis 15 ml). Pubertal Tanner staging revealed axillary hair stage 3, pubic hair stage 5, and no gynecomastia. At the age of 17 years, the patient received a 250 mg testosterone injection every 2 weeks. Hormonal assessment revealed **FSH** 69.45 mIU/ml (normal value: 2.9–8.2 mIU/ml) and **LH** 57.67 mIU/ml (normal value: 1.8–5.2 mIU/ml). Gonadal biopsy is not available for ethical reasons.

### Exome sequencing

The *AR* and *SRD5A2* genes’ sequencing analyses revealed wild-type sequences; as a result, WES was indicated in order to pinpoint the causal gene variant. Out of the 127,091 variants obtained from exome sequencing of the patient, potentially pathogenic variants related to the patient’s phenotype were selected. Variants with minor allele frequencies (MAF > 0.05) were filtered out during the first stage of analysis. Three gene variants were found to be related to the patient’s phenotype (Supplementary Table 1). Only the LHCGR variants were further evaluated since the AMH and WT1 gene variants were classified as benign by ACMG guidelines and ClinVar. Two heterozygous variants of uncertain significance in the LHCGR gene (c.55_56insTGCTGAAGCTGCTGCTGCTGCTGCAGCTGCAGC and c.1331T > G) are considered to be potentially pathogenic.

### Confirmatory Sanger sequencing

**Fig. 1:**
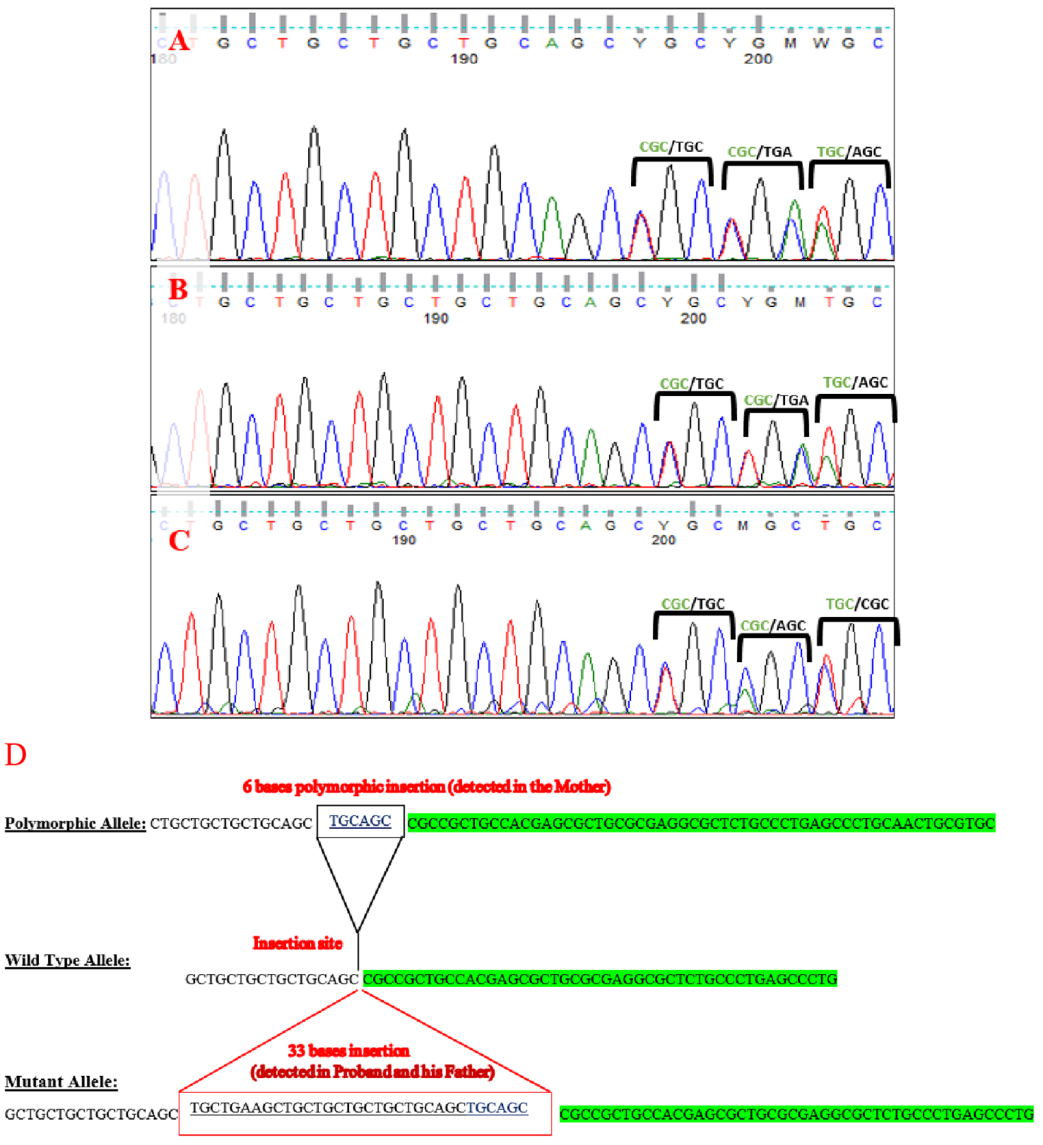
Sequence chromatogram for exon 1 of the *LHCGR* gene showing the site of the insertion mutation. (A) the patient’s sequence, (B) the father’s sequence with heterozygous insertion of 33 bases c.55_56insTGCTGAAGCTGCTGCTGCTGCTGCAGCTGCAG, and (C) the mother’s sequence with heterozygous 6 bases insertion NM_000233.4:c.55_56insTGCAGC. The sequence of the two overlapping alleles (heterozygous insertion) is indicated above the sequence (the wild-type in green and the mutant in black). (D) The three detected alleles in exon 1 of the proband and his parents. The proband and his father had the wild type allele and the mutant allele, while the mother had the wild type allele and the polymorphic allele

LHCGR gene variants underwent Sanger sequencing of exons 1 and 11 in the patient and his parents. The results showed compound heterozygous variants in the patient (Fig. 1).

Exon 1 insertion 33-bases variant (c.55_56insTGCTGAAGCTGCTGCTGCTGCTGCAGCTGCAGC) (p.Gln18_Pro19insLeuLeuLysLeuLeuLeuLeuLeuGlnLeuGln) was found in a heterozygous state in the patient and his father. The 33-bases insertion variant was previously reported as a pathogenic cause of LCH type I [24, 36].

Sequencing of exon 1 of the mother revealed heterozygous insertion of 6-bp (NM_000233.4:c.55_56insTGCAGC) (p.Leu17_Gln18dup), which is a common polymorphic insertion in Caucasian populations.

The paternally inherited 33-bases variant is composed of a 27-bp insertion of the (TGC TGA AGC TGC TGC TGC TGC TGC AGC) sequence and a polymorphic insertion of 6-bp TGC AGC (Leu Gln variant) commonly found in Caucasians [26].

**Fig. 2:**
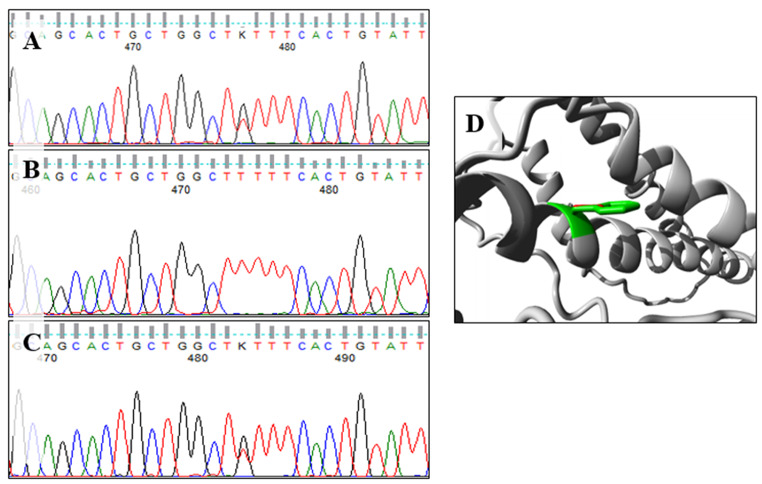
The detected missense variant in exon 11 (c.1331T > G; p.Phe444Cys). Sequence chromatogram for exon 11 of *LHCGR* gene showing the site of the variant **(A)** in the patient with heterozygous pattern, **(B)** in the father with wild-type sequence, and **(C)** in the mother with the heterozygous form. **(D)** A ribbon presentation of the protein by HOPE project showing the side chains of the wild-type and mutant residues, which are colored green and red, respectively

The inherited maternal allelic variation was a missense variant NM_000233.4:c.1331T > G. (p.Phe444Cys) detected in exon 11. There was no record of the variant in any exome or genome sequencing database or project, e.g., GnomAD, dbSNP, or 1000 Genomes. The applied in-silico prediction tools showed high pathogenicity scores in most of them. The indicative predication was damaging and deleterious by FATHMM, LRT, M-CAP, MutPred, Polyphen-2, SIFT, and PROVEAN. High scores (> 0.9) with a pathogenic interpretation were revealed by REVEL and MetaRNN. The predicted ΔΔG (kcal/mol) estimated by SDM, CUPSAT, and DUET tools were − 1.06, -1.34, and − 0.053, respectively, which revealed that the mutant protein was destabilizing and had reduced protein stability. The mutated residue (p.Phe444Cys) is located in the third TMD and is in contact with the disulfide bond formed between the first and second extracellular loops. The variant could potentially disrupt the interplay between these domains, leading to a possible impact on the protein’s function. Moreover, the mutant amino acid cysteine exhibits reduced size when compared to the wild-type amino acid phenylalanine, thereby generating a void in the protein’s core (Fig. 2).

## Discussion

LCH type II is a rare subtype of LCH. Although it was first described in 1985 [30], few reports have described patients with partial (insufficient) *LHCGR* activation in 46,XY patients [1, 8, 9, 11, 12, 34]. Most of the mutant proteins causing LCH type II produced partially active *LHCGR*, which retain some responsiveness to LH and hCG hormones, implying normal male development during fetal life. After birth, patients were presented with micropenis, cryptorchidism, and/or hypospadias and sometimes only infertility [8, 12, 16]. In contrast, LCH type I is the more severe form associated with complete inactivation of LHCGR, which causes complete unresponsiveness to LH and hCG hormones. This results in 46,XY patients with female external genitalia, mostly diagnosed at puberty due to primary amenorrhea [22, 35].

LCH type II is caused by *LHCGR* gene variants which occur in homozygous or compound heterozygous status. All previously reported cases, to the best of our knowledge, had at least one variant in exon 11 or exon 10. These patients had micropenis, as well as hypospadias and cryptorchidism, which were not present in all cases. Testosterone levels were low or undetectable. Serum LH and FSH levels were diverse, ranging from normal to slightly elevated ranges. Furthermore, the hCG stimulation test results ranged from being unresponsive or only partially responsive to being responsive and reaching normal levels [1, 8, 9, 11, 12, 34]. Contrary to LCH type I, serum gonadotropins were significantly elevated (Supplementary Table 2).

In the current study, the phenotype is consistent with the previously reported ones, especially the patient reported by Vezzoli and colleagues [34]. The latter patient had compound heterozygous missense variants in exons 1 and 11. Both patients had low testosterone levels with poor responses to the hCG stimulation test and slightly elevated serum LH and FSH levels.

**Fig. 3:**
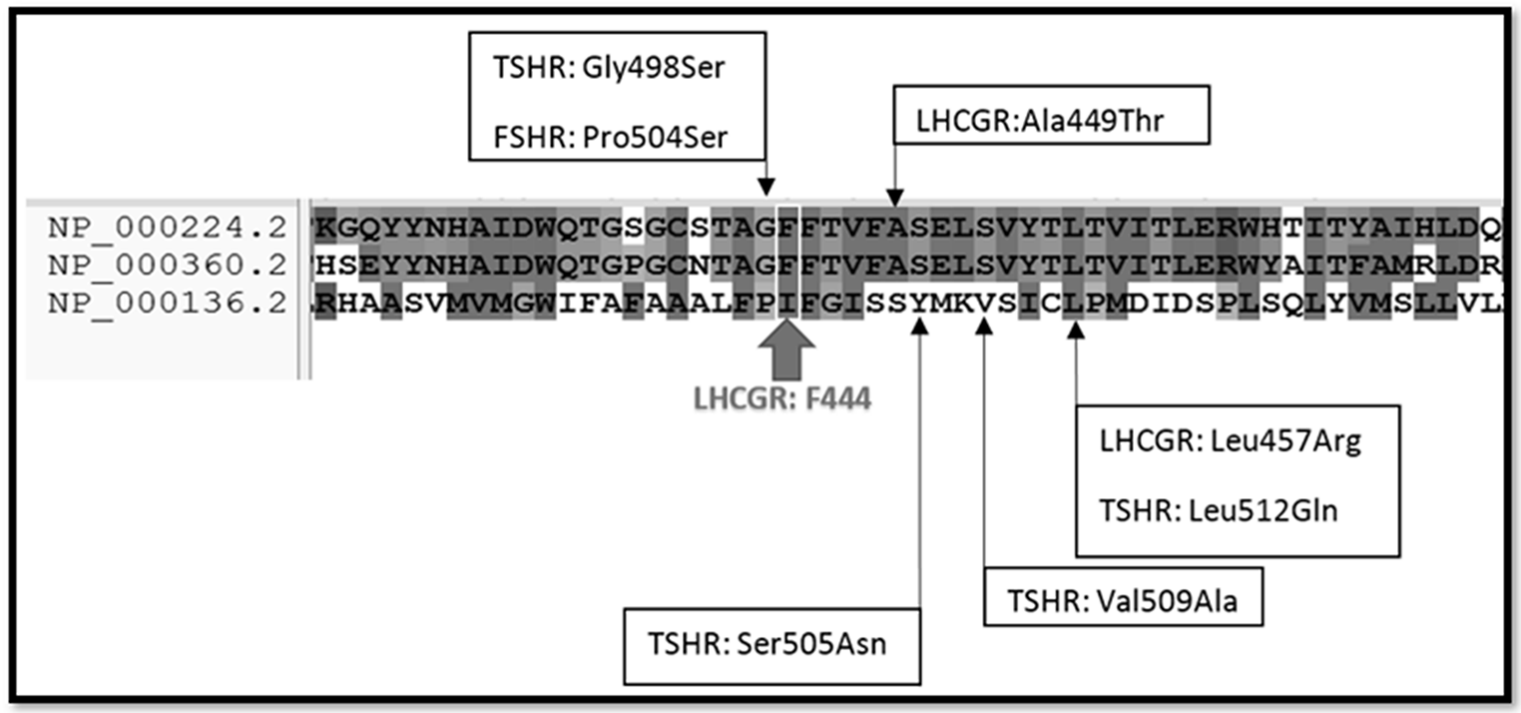
Multiple sequence alignment of *LHCGR* (NP_000224.2), *TSHR* (NP_000360.2), and *FSHR* (NP_000136.2) proteins sequences using ClastalX2. The big arrow points to the site of the detected variant in the current study. Small arrows indicate the site of reported variants (variants in the upper section are inactivating causing thyroid-stimulating hormone resistance, resistant ovarian syndrome, and empty follicle syndrome, while variants in the lower section are activating ones, resulting in hyperthyroidism and male precocious puberty)

Reported variants related to LCH type II, as shown in Supplementary Table 2, were located within the seventh TMD (p.Ser616Tyr and p.Ile625Lys) or the second TMD (p.Ile415Thr) [8, 9, 11, 12, 34]. Herein, the third TMD (440–462 aa) with the detected missense variant (p. Phe444Cys) has broadened the genetic variation spectrum. Phenylalanine 444 of the LHCG hormone receptor is an exclusively conserved site within different species. Moreover, it is conserved within the G protein-coupled receptors (Fig. 3). On the other hand, the mutant amino acids located around Phe444 showed a loss of function (five residues up- and downstream) in either LHCGR, TSHR, or FSHR proteins [6, 17, 37]. Moreover, protein stability prediction tools also confirmed decreased stability of the protein due to the effect of this variation. The aforementioned causes outweigh the pathogenic effect of the variant.

The human LHCGR’s signal peptide comprises the first 26 amino acid residues. The signal peptide, particularly the central area, is necessary for the trafficking of precursor proteins from the ribosomes to the endoplasmic reticulum, whereas the C-terminus of the signal peptide contains polar amino acids such as glycine or proline, which are required for cleaving the protein [27]. The signal peptide cleavage site is ahead of the 33-bases insertion. As a result, it may interfere with its function because it lengthened the 26-amino acid peptide to 37 amino acids [14]. In vitro cell expression of the insertion mutant receptor has shown the complete absence of its function; the mutant receptor did not bind the ligand on the cell surface or in cell lysates, which indicates that either a binding or a trafficking defect occurred [36]. To the best of our knowledge, six patients were reported with the insertion variant either in homozygous or compound heterozygous patterns (Supplementary Table 3). Interestingly, all the patients presented with Leydig cell hypoplasia type I and had female external genitalia [10, 21, 24, 28, 36]. Charmandari and colleagues described a case of LCH type I in which the signal peptide insertion variant coexisted with a homozygous p.Gly71Arg variant. Although parental segregation wa*s* not available since the patient was adopted, experimental studies verified the pathogenicity of the missense variant [4]. This study is the first to report a patient with the 33-bases insertion variant, who presented with LCH type II. Homozygous insertion of either 27 or 33-bases had a severe effect on the protein function, which resulted in retention in the endoplasmic reticulum and receptor degradation [32]. It is of note that the previously reported patients with compound heterozygous variants had either stop-gain in the 4th and 5th TMDs or signal peptide deletion frameshift allelic variants, which would explain the severity of the phenotype [10, 21, 24, 36].

The gonad histopathology findings appeared to be comparable for both types (LCH I and LCH II). Leydig cells were scarce and revealed hypoplasia or possibly aplasia (Supplementary Tables 2 & 3). This results from either the inability of Leydig cell precursors to mature or the lack of Leydig cell differentiation. The histological picture of LCH type II patients showed either lack of mature Leydig cells or absence of Leydig cells, which could be due to variability in the receptor response, studied biopsy or excised gonads, and the rarity of cases [5, 29, 34].

In the current study, the less severe subtype (LCH type II) is presented, which is contributed by the missense variant in the third TMD along with the 33-bases insertion variant of the signal peptide. Variants of the TMDs have variable effects, ranging from misfolding and impaired trafficking to coupling and/or signaling efficacy impairment. To the best of our knowledge, the third TMD has not previously been associated with any known variants in the LHCGR gene. On the other hand, the codon prior to the mutant Phe444 of LHCGR in the TSHR protein is p.Gly498Ser (Fig. 3). The latter variant reduced TSH binding, cAMP response to ligand, and cell surface expression despite normal biosynthesis of the TSHR receptor [17]. In silico functional studies have shown that the p.Phe444Cys variant would affect the mutant LCHGR protein folding, and also coupling/signaling efficacy, since cysteine residues can form disulfide bonds with other cysteine residues and potentially disrupt the proper folding of the protein. Additionally, the substitution of phenylalanine with cysteine can affect the hydrophobic interaction within the TMD. This could lead to impaired trafficking of the receptor to the cell surface and ultimately affect its function in responding to its ligand. The detected novel missense variant (p.Phe444Cys) reduced the severe effect of the insertion variant, resulting in a milder phenotype of our patient.

## Conclusion

Most variants in the signal peptide have severe effects, resulting in retention in the endoplasmic reticulum and receptor degradation, and are associated with LCH type I. However, we described a patient with LCH type II due to compound heterozygous variants, one in the signal peptide and a novel missense variant in the third TMD. Therefore, identifying other variants in the TMDs of LHCGR, specifically the third, could provide valuable insights into the molecular mechanisms underlying LCH, infertility, and other related conditions. Experimental functional studies are recommended to confirm the pathogenic effect of the novel variants, either a partial or a total inactivating outcome. Additionally, this broadens the phenotype spectrum related to the 27 or 33-bases insertion variant of the signal peptide and highlights the importance of considering variants beyond the signal peptide in understanding the pathogenesis of LCH.

### Electronic supplementary material

Below is the link to the electronic supplementary material.

Supplementary Material 1

Supplementary Material 2

Supplementary Material 3

## Data Availability

The data that support the findings of this study are available from the corresponding author upon reasonable request.
