# Supplementary material for: Expanding the phenotypic spectrum of LHCGR signal peptide insertion variant: novel clinical and allelic findings causing Leydig cell hypoplasia type II
Source: Hormones (Athens). 2024 Mar 25;23(2):305–12. doi: 10.1007/s42000-024-00546-x (PMC11219444; doi:10.1007/s42000-024-00546-x)
Supplement: Supplementary file 1 — Supplementary Material 1 [file 42000_2024_546_MOESM1_ESM.docx]

**Table 1: Candidate Gene Variants identified by exome sequencing potentially related to patient's phenotype**

| *Gene* | **Detected variant** | **Zygosity**  **& MAF** | **ClinVar** | **MetaRNN** | **REVEL** | **ACMG classific** |
| --- | --- | --- | --- | --- | --- | --- |
| *AMH* | NM_000479.5:c.974A>G  (p.Gln325Arg)  rs140765565 | Homozygous  0.009664 (1352/139900, GnomAD) | Benign/Likely benign​ in 5 submissions (VCV000381531.14) | 0.009387 (Benign) | 0.425 (Benign) | Benign  (BP6, BS1, BS2, BP4, BP1) |
| *WT1* | NM_024426.6:c.216G>T  (p.Gln72His)  rs5030135 | Heterozygous  0.000408 (62/151948, GnomAD) | Benign/Likely benign​ in 14 submissions.  (VCV000241479.18) | 0.002192  (Benign) | 0.054  (Benign) | Benign  (BS1, BS2, BP4, BP6) |
| *LHCGR* | NM_000233.4:c.1331T>G  (p.Phe444Cys) | Heterozygous  (no frequency found) | No data available | 0.9708  (Pathogenic) | 0.947  (Pathogenic) | VUS  (PP3, PM1, PM2) |
| *LHCGR* | NM_000233.4:c.50_55dup  (p.Leu17_Gln18dup)  rs71245621 | Heterozygous  0.189* (25112/132330, GnomAD) | Benign​ in 8 submissions.  (VCV000336471.9) | - | - | Benign  (BA1, BP3, BP4, BP6, PM4) |
| *LHCGR* | NM_000233.4:c.55_56insTGCTGAAGCTGCTGCTGCTGCTGCAGCTGCAGC  (p.Gln18_Pro19insLeuLeuLysLeuLeuLeuLeuLeuGlnLeuGln) | Heterozygous  0.0000151 (2/132330, GnomAD) | Pathogenic in 1 submission.  (VCV000014405.1) | - | - | VUS  (PM4, PM2, PP5, BP4) |

* This variant is added to the table because its overlapping position with anther detected variant.
