# Supplementary material for: Expanding the phenotypic spectrum of LHCGR signal peptide insertion variant: novel clinical and allelic findings causing Leydig cell hypoplasia type II
Source: Hormones (Athens). 2024 Mar 25;23(2):305–12. doi: 10.1007/s42000-024-00546-x (PMC11219444; doi:10.1007/s42000-024-00546-x)
Supplement: Supplementary file 2 — Supplementary Material 2 [file 42000_2024_546_MOESM2_ESM.docx]

|  | **Laue et al., 1996** | **Martens et al., 1998** | **Latronico et al., 1996** | **Kossack et al., 2013** | **Vezzoli et al., 2015** | **Alla et al., 2022** |
| --- | --- | --- | --- | --- | --- | --- |
|  | **Compound heterozygous**  c.872A>G  p.Asn291Ser (Exon 10) | **Homozygous**  c.1874T>A  p.Ile625Lys (Exon 11) | **Homozygous**  c.1847C>A p.Ser616Tyr (Exon 11) | **Compound heterozygous**  c.580A>G  p.Lys194Glu (Exon 6A) | **Compound heterozygous**  c.1847C>A p.Ser616Tyr (Exon 11) | **Homozygous**  deletion of approximately 4 bp (Exon 10) |
|  | c.1847C>A  p.Ser616Tyr (Exon 11) |  |  | c.1244T>C p.Ile415Thr (Exon 11) | c.29C>T  p.Leu10Pro (Exon 1) |  |
| **Age at diagnosis** | 2.3 years | 3 brothers 28y, 35y & 51y | at birth | at birth | at birth | 11 years 7 months and 1 year 6 months |
| **Karyotype** | 46,XY | 46,XY | 46,XY | 46,XY | 46,XY | 46,XY |
| **External genitalia** | The length of the phallus was only 2 cm with severe perineoscrotal hypospadias. Each gonad was in the upper canal and 1 ml in volume. | Micropenis, absence of pubertal signs, and infertility. | The length of his stretched phallus was 1.5 cm (more than 2.5 SD below the normal mean for age). Both testes were descended, with a volume of approximately 1 ml each. | Micropenis, left testis was located in the upper scrotal compartment, the right gonad was not palpable initially but could be located in the inguinal region at 7 months of age. | Micropenis, cryptorchidism and hypospadias. | Micropenis and cryptorchidism. |
| **hCG stimulation test** | non responsive | poor response | non responsive | non responsive | poor response | respond to reach normal values |
| **Histopathology of gonads** | Immature seminiferous tubules without Leydig cells. | Seminiferous tubules and lacked mature Leydig cells. | NA | NA | Leydig cells are not visualized, and seminiferous tubules  with a thick basal membrane and Sertoli cells can be seen without evidence of germinal cells. | - |
| **Testosterone** | 0.1 ng/ml | 0.84 ng/ml | <0.1 ng/ml | <0.1 ng/ml | <0.2 ng/ml | - |
| **LH (IU/ml)** | 1 mIU/ml | (↑) | 6.3 mIU/ml | normal | 10.9 mIU/ml | - |
| **FSH (IU/ml)** | 3 mIU/ml | (↑) | 1.3 mIU/ml | normal | 6.5 mIU/ml | - |

**Table 2: Clinical, hormonal, and molecular results of the previously reported LCH type II cases.**
