# Supplementary material for: Expanding the phenotypic spectrum of LHCGR signal peptide insertion variant: novel clinical and allelic findings causing Leydig cell hypoplasia type II
Source: Hormones (Athens). 2024 Mar 25;23(2):305–12. doi: 10.1007/s42000-024-00546-x (PMC11219444; doi:10.1007/s42000-024-00546-x)
Supplement: Supplementary file 3 — Supplementary Material 3 [file 42000_2024_546_MOESM3_ESM.docx]

**Table 3: Clinical, hormonal, and molecular results of the previously reported cases with the insertion mutation in exon 1 of LHCGR gene**

|  | **Laue et al., 1995 and Wu et al., 1998** | **Laue et al., 1995 and Wu et al., 1998** | **Richter-Unruh et al., 2002** | **Sinha et al., 2011** | **Sinha et al., 2011** | **Potorac et al., 2019** |
| --- | --- | --- | --- | --- | --- | --- |
|  | Compound hetero  33-bp insertion in exon 1/p.Cys545Ter in exon 11 [5TMD] | Compound hetero  33-bp insertion in exon 1/p.Cys545Ter in exon 11 [5TMD] | Compound hetero  33-bp insertion in exon 1/p.Try491Ter in exon 11 [4TMD] | Homozygous  27-bp insertion in exon 1 | Homozygous  27-bp insertion in exon 1 | Compound hetero  27-bp insertion in exon 1/ p.Lys12_Leu15del in exon 1 |
| **Age at diagnosis** | 6.5 years | 4.5 years | 17 years | 16m | 2 days | 16 years |
| **origin** | Not available | Not available | Netherlands | Lebanon | Lebanon | Caucasian |
| **Parents consanguinity** | no | no | no | 1^st^ cousin | 1^st^ cousin | Not available |
| **Karyotype** | 46,XY | 46,XY | 46,XY | 46,XY | 46,XY | 46,XY |
| **External genitalia** | female | female | female | female | female | female |
| **hCG stimulation test** | unresponsive | unresponsive | Not performed | unresponsive | unresponsive | Not performed |
| **Histopathology of gonads** | Absence of Leydig cells | Absence of Leydig cells | Absence of Leydig cells | Not performed | Not available | Leydig cell hypoplasia |
| **Testosterone** | 0.06 ng/ml | 0.14 ng/ml | 0.32 ng/ml | 0.06 ng/ml | Not available | 0.17 ng/ml |
| **LH** | Not available | Not available | 14.1 mIU/ml | 0.8 mIU/ml | Not available | 32.6 mIU/ml |
| **FSH** | Not available | Not available | 4.9 mIU/ml | 2.74 mIU/ml | Not available | 6.6 mIU/ml |
